# Intercellular Communication by Exchange of Cytoplasmic Material via Tunneling Nano-Tube Like Structures in Primary Human Renal Epithelial Cells

**DOI:** 10.1371/journal.pone.0021283

**Published:** 2011-06-27

**Authors:** Sophie Domhan, Lili Ma, Albert Tai, Zachary Anaya, Afshin Beheshti, Martin Zeier, Lynn Hlatky, Amir Abdollahi

**Affiliations:** 1 Department of Medicine, Center of Cancer Systems Biology, St. Elizabeth's Medical Center, Tufts University, Boston, Massachusetts, United States of America; 2 Department of Radiation Oncology, Molecular RadioOncology, Heidelberg Ion Therapy Center (HIT), University of Heidelberg Medical School and National Center for Tumor Diseases (NCT), German Cancer Research Center (DKFZ), Heidelberg, Germany; 3 Department of Nephrology, University of Heidelberg Medical School, Heidelberg, Germany; Medical College of Georgia, United States of America

## Abstract

Transfer of cellular material via tunneling nanotubes (TNT) was recently discovered as a novel mechanism for intercellular communication. The role of intercellular exchange in communication of renal epithelium is not known. Here we report extensive spontaneous intercellular exchange of cargo vesicles and organelles between primary human proximal tubular epithelial cells (RPTEC). Cells were labeled with two different quantum dot nanocrystals (Qtracker 605 or 525) and intercellular exchange was quantified by high-throughput fluorescence imaging and FACS analysis. In co-culture, a substantial fraction of cells (67.5%) contained both dyes indicating high levels of spontaneous intercellular exchange in RPTEC. The double positive cells could be divided into three categories based on the preponderance of 605 Qtracker (46.30%), 525 Qtracker (48.3%) and approximately equal content of both Qtrackers (4.57%). The transfer of mitochondria between RPTECs was also detected using an organelle specific dye. Inhibition of TNT genesis by actin polymerization inhibitor (Latrunculin B) markedly reduced intercellular exchange (>60%) suggesting that intercellular exchange in RPTEC was in part mediated via TNT-like structures. In contrast, induction of cellular stress by Zeocin treatment increased tube-genesis in RPTEC. Our data indicates an unexpected dynamic of intercellular communication between RPTEC by exchange of cytosolic material, which may play an important role in renal physiology.

## Introduction

Intercellular communication is a key process in the development and maintenance of multicellular organisms. The classical type of intercellular communication is attributed to cell-membrane surface based receptor-ligand interactions. However, other intercellular structures such as gap junctions and synapses have been described to mediate cell–cell communication via exchange of cellular content in e.g. human heart muscle cells and neurons.

Recently, a novel mechanism for intercellular communication was discovered by which nanotubular structures, consisting of thin membrane bridges, mediate membrane continuity between mammalian cells [1]. These channels, referred to as tunneling nanotubes (TNT), were shown to actively traffic cytosolic content from cell to cell within the interior of their filaments [1]. Among the proposed functions of TNTs are the exchange of endosome-related organelles and other cellular components over long distances, and the coordination of signaling between the connected cells [1]–[3]. Calcium ions, major-histo-compatibility proteins (MHC class I), prions, viral and bacterial pathogens, small organelles of the endosomal/lysosomal system and mitochondria are among the hitherto identified TNT cargos [3]–[7]. TNTs were first described in cultured rat pheochromocytoma PC12 cells [1] and have been subsequently shown to connect a growing number of cell types [2]. The emergence of the first *in-vivo* evidence for TNT-connectivity between immune cells of the corneal stroma suggest a central role for TNT-based intercellular communication in physiological processes of multi-cellular organism [8].

Here we report extensive spontaneous exchange of cellular material between human renal proximal tubular epithelial cells (RPTEC). The study of intercellular exchange in RPTECs was motivated by our incidental observation of TNT-like structures bridging primary epithelial cells in culture. Intercellular exchange via TNT based cell-communication was reported in cells that are characterized by high motility and plasticity e.g. in progenitor cells, immune cells or tumor cells [2]. In contrast, the role of intercellular exchange of cellular material in communication of human epithelial cells that display tight intercellular junctions has not been explored. Therefore, our data point towards a new direction of research by better understanding the role of intercellular exchange in communication of renal epithelium.

## Results

### Identification of TNT-like structures in epithelial cells

**Figure 1:**
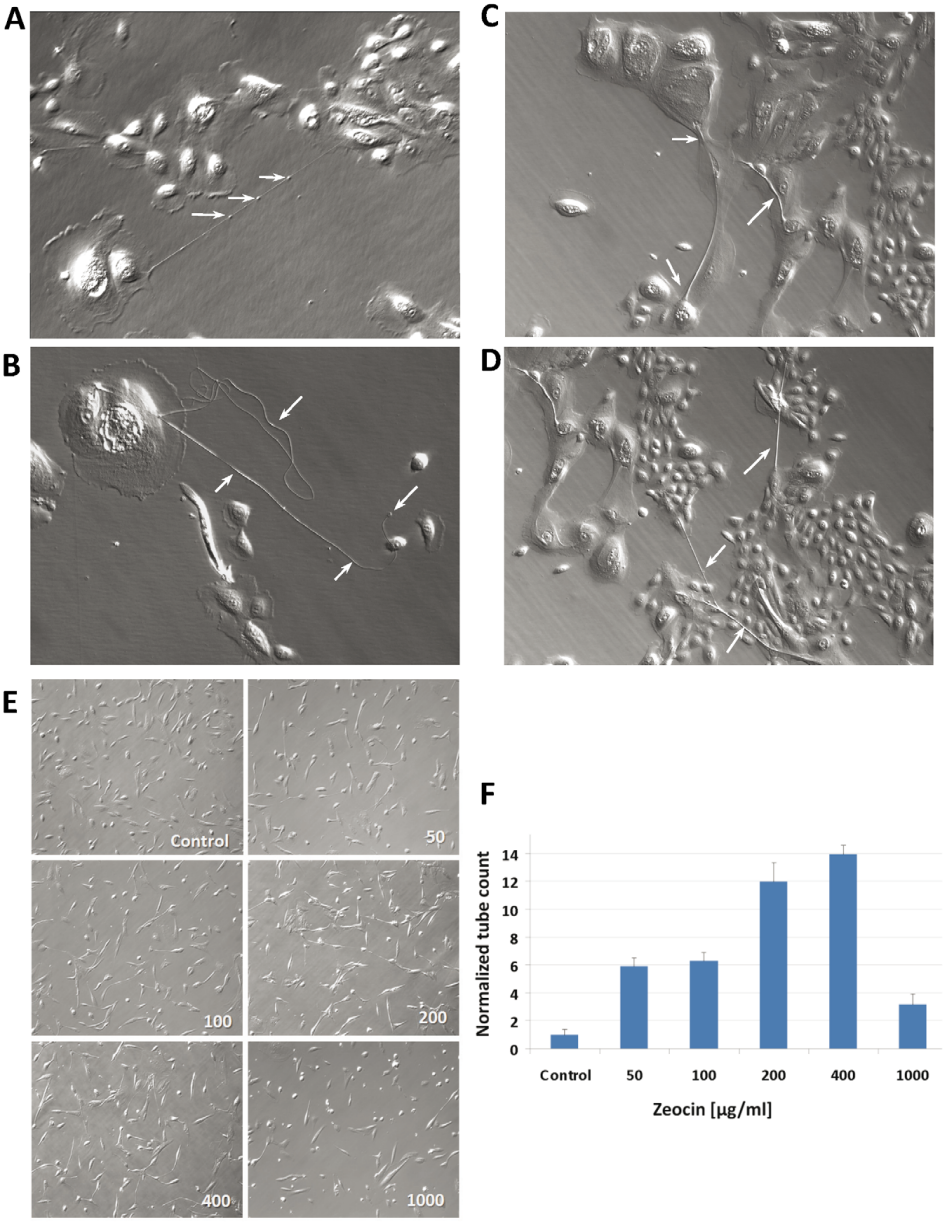
TNT-like tubes connecting epithelial cells. Representative phase contrast images of the identified TNT-like tubes connecting HMECs. These membranous structures were able to span long distances of several micrometers between the cells (A–D). The observed “gondola”-like structures, i.e., localized changes in tube diameter (white arrows in (A)), indicated potential transfer of cargos in the lumen of the tubes. Tubes with blind ends were also found (white arrow in (B)). In contrast to TNTs the identified membranous tubes often build cross roads bridging several cells with each other (C). A frequent observation was the connection of epithelial cell “islands” via these tubes (C and D). All photomicrographs x100 view. **Enhanced tube-genesis followed by cellular stress** (E and F). Treatment of RPTEC with the glycopeptide antibiotic *Zeocin* led to a marked increase in the number of tubes in a dose dependent manner. The maximum induction of tubes was reached at 400 µg/ml dose of *Zeocin*. At higher concentrations (e.g. 1000 µg/ml) the toxicity and cell death effects of the treatment dominated the phenotype resulting in a decline of the number of tubes and vital cells per optical field. Bars represent means ± *SD* from quadruplicates measurements (p<0.01 for 50–400 µg/ml *Zeocin* treatment vs. control).

**Figure 2:**
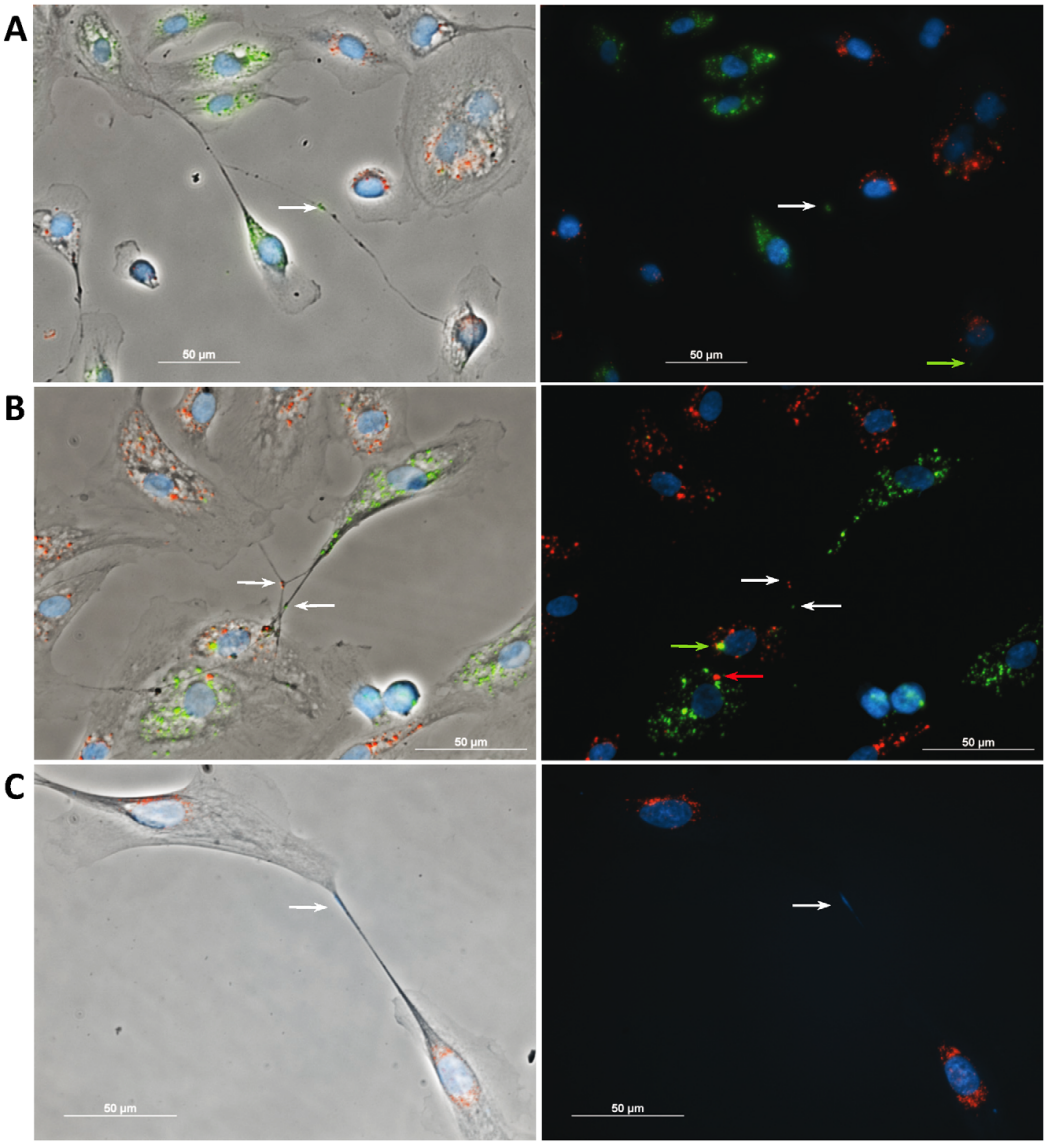
Tracing intercellular exchange by Qdot® nanocrystals. (**A–C**)**,** RPTEC were labeled with either Qtracker 605 (red) or 525 (green) and co-cultured for 24 h. To-Pro-3 was used to stain the cell nucleus (DNA, blue). Fluorescence microscopy revealed a number of “double positive” cells suggesting spontaneous intercellular exchange of cytoplasmic material between the RPTECs. Fluorescence particles were also detected in the lumen of the tubular structures (arrows) indicating endoluminal transport. Among the specific morphological features of RPTEC tubes as compared to classical TNTs are their larger caliber, their ability to bridge very long distances (>200 µm) and the more frequent appearance of bifurcations or connections of multiple cells (A and B). Occasionally To-Pro-3 positive DNA-signal (blue staining) was detected in the lumen of the tubes (C) suggesting the possibility of the exchange of genetic material between RPTEC.

Using light microscopy, thin membranous structures bridging two or more cells were detected in human renal proximal tubular epithelial cells (RPTEC) and human mammary epithelial cells (HMEC) (Figure 1). These non-transformed primary isolated cells display characteristic epithelial tissue architecture i.e., a cobble stone morphology with tight cell-cell junctions in culture. Membranous cell-cell connections were able to span long distances of >200 µm between the cells and were often connecting two distant epithelial “cell- islands” (Figure 1). We also found blindly ending filopodia- like cell protrusions and branched connections between three or more cells (Figures 1 and 2). The caliber of the membranous tubes ranged from 200 nm to >1 µm. Localized and multiple distensions along the tubes forming gondola-like structures indicated potential involvement of these tubes in intercellular transport (Figure 1A). The identified tubes demonstrated low photo-sensitivity and were visible under light microscopy as stable structures up to several minutes.

### Increased tube-genesis by cellular stress

To explore the role of cellular stress on intercellular communication, RPTECs were exposed to various Zeocin concentrations (from 50–1000 ng/ml). Zeocin is a copper-chelated glycopeptide antibiotic that causes cell death by intercalation and cleavage of DNA. We found a dose dependent induction of tube-genesis with a maximum of 10-Fold increase in tube numbers after incubation of RPTECs with 400 ng/ml Zeocin (Figure 1E and F). Hence, the enhanced formation of tubes might constitute a coordinated process facilitating intercellular communication between RPTECs under stress conditions. At the highest administered Zeocin dose (1000 ng/ml) the number of RPTEC tubes declined together with the viability of the cells due to treatment related toxicity.

### Tracing intercellular exchange by quantum dot nanocrystals

To detect potential exchange of the cytosolic content, RPTECs were separately labeled with two distinct fluorescent quantum dot nanocrystals (Qdots, Qtracker®). The Qdots have narrow emission peaks and are internalized in live cells providing intense, stable fluorescence at the corresponding wavelengths that can be traced through several generations by fluorescence microcopy or FACS analysis. Qtracker 605 consists of orange fluorescent particles whereas Qtracker 525 provides green fluorescence. After labeling of cells with Qtracker 605 or 525, respectively, cells were co-cultured under standard culture conditions for 24 h. We found extensive spontaneous exchange of Qdots between the RPTECs as detected by numerous “double positive” cells by fluorescence microscopy (Figure 2). Quantum dot particles could also be detected in the lumen of the intercellular bridges (Figure 2) indicating intraluminal transport. However, the dynamic of tube genesis and intercellular exchange of the Qdots was much slower in RPTEC and HMEC tubes compared to the classical TNTs i.e., the exchange of Qdots could not be traced within 30 sec-5 min of observation period as determined by spinning disk confocal time lapse microscopy (data not shown). Longer observation periods were limited due to the high sensitivity of the primary epithelial cells to the phototoxic effects of the laserscans.

### The dynamic and extent of intercellular transfer

**Figure 3:**
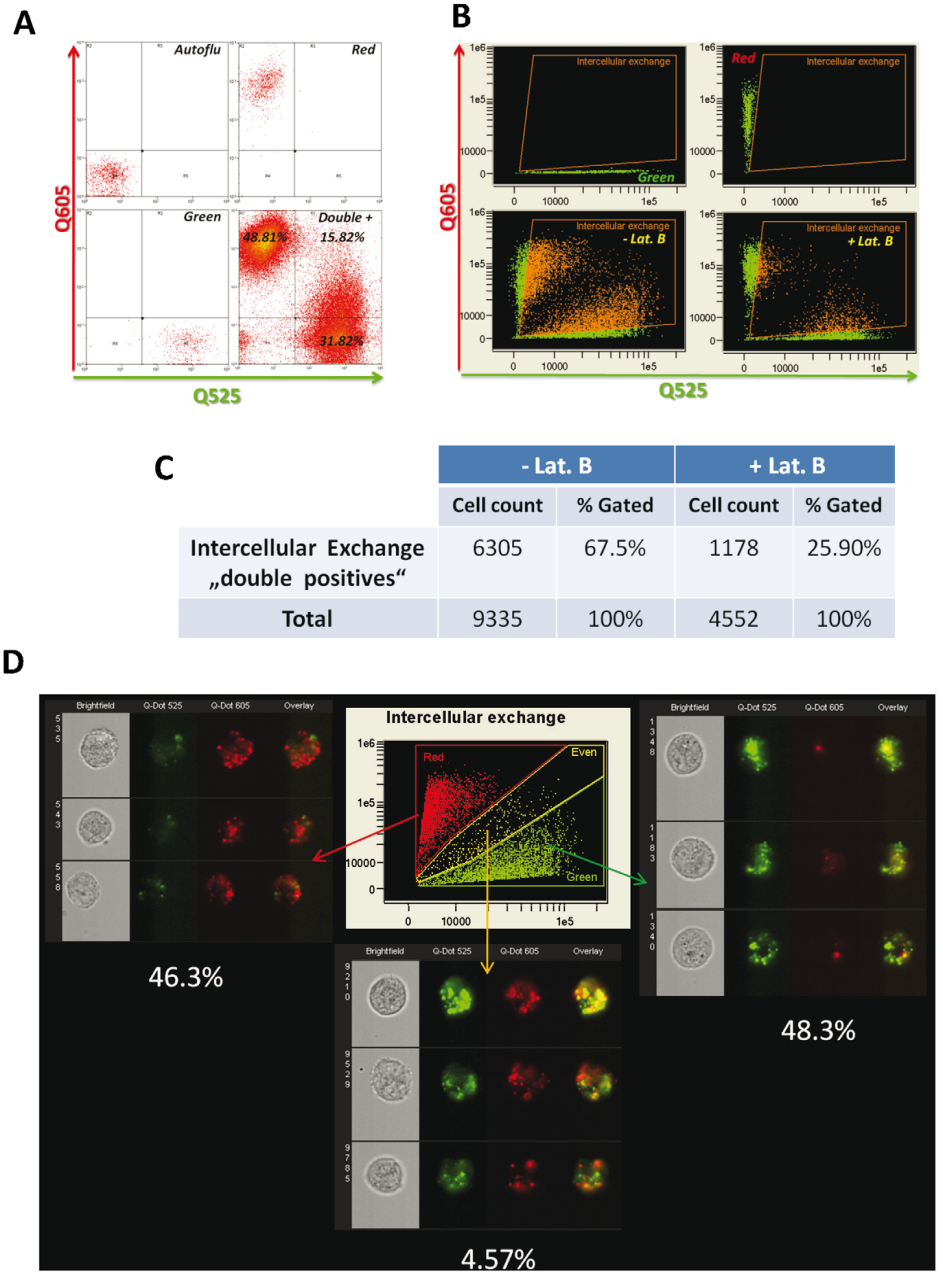
Quantifying the spontaneous intercellular exchange rates of organelles in epithelial cells. Qtracker 605 and 525 labeled cells were co-cultured for 24 h and analyzed by flow cytometry (A). FACS analysis revealed 15.8% “double +” cells. To overcome the limitation of FACS analysis to detect and discriminate double positive cells with a low number of transfered Qdots by the tubes, we employed high-throughput fluorescence image analysis using the ImageStream™ platform (B–D). By analyzing 9335 co-cultured RPTEC, spontaneous intercellular exchange was detected in 67.5% or 6305 cells (B–C). Inhibition of tube-genesis by Lat. B resulted in marked (62%) reduction of the intercellular exchange (from 67.5% to 25.9% double positive cells). Detailed analysis revealed that the intercellular exchange resulted in three categories of double positive cells: i) transfer of 525 Qtracker to 605 Qtracker labeled cells (46.30% or 2921 cells), ii) transfer of 605 Qtracker to 525 Qtracker labeled cells (48.3% or 3046 cells) or equal content of both Qtrackers (4.57% or 288 cells) (D). Thus, the transfer of few labeled Qdots organelles contributed to the majority of spontaneous intercellular exchange among RPTEC.

To quantitatively analyze the extent of intercellular exchange, Qtracker 605 and 525 labeled epithelial cells were co-cultured for 24 h and analyzed by flow cytometry (Figure 3A). Using a stringent cut off criteria, ∼15.8% of HMEC were found to be “double positive” by FACS analysis, i.e., contain both 605 and 525 Qdots after co-culture (Figure 3A). To accurately determine even minimal exchange of quantum dot positive organelles, we performed high-throughput fluorescence image analysis using the ImageStream™ platform. Bright field- combined with two channel fluorescence microscopy of 9335 co-cultured RPTEC revealed that spontaneous intercellular exchange occurred in a substantial fraction of renal epithelial cells (67.5% or 6305 double positive cells, Figure 3B). These data indicate extensive spontaneous intercellular exchange in RPTEC. Detailed analysis of “double positive” cells revealed that they could be divided into three categories based on the preponderance of 605 Qtracker (46.30% or 2921 cells), 525 Qtracker (48.3% or 3046 cells) and approximately equal content of both Qtrackers (4.57% or 288 cells) (Figure 3D). These data suggest that unidirectional transport may constitute the predominant pattern of exchange in RPTECs.

### Inhibition of tube-genesis markedly impaired intercellular exchange

Actin polymerization is essential for development of TNTs. Hence, inhibitors of F-actin polymerization such as Latrunculin-B (LatB) were used to confirm the relevance of TNT-genesis in intercellular communication [1]. Therefore, to determine the role of TNT-like structures in intercellular exchange, 605 and 525 labeled RPTEC co-cultures were investigated in presence vs. absence of Latrunculin B (1.25 µM). TNT formation was substantially reduced after LatB treatment. ImageStream analysis of 4552 co-cultured RPTECs revealed a 62% reduction in the number of double positive cells after LatB treatment (25.9% or 1178 cells) as compared to the non-treated control (Figure 3B and C). These data suggest that tube-based intercellular exchange plays a critical role in communication between RPTECs.

### Exchange of mitochondria between RPTECs

**Figure 4:**
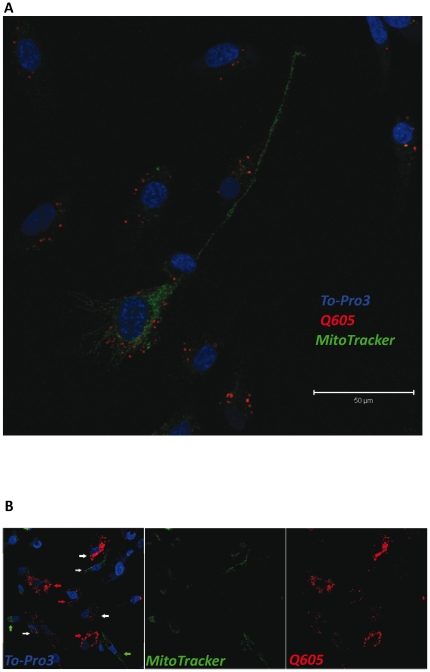
Intercellular exchange of Mitochondria. To detect potential exchange of other organelles such as mitochondria between RPTECs, cells were labeled with MitoTracker® reagent which specifically labels active mitochondria *in-vivo* (green). MitoTracker labeled cells were co-cultured for 24 h together with Qtracker 605 (red) labeled cells. Mitochondria could be detected in the lumen of the TNT-like tubes in RPTEC (A). The exchange of organelles between RPTEC is shown (highlighted by white arrows). Q605 labeled cells (red arrow). MitoTracker labeled cells (green arrow). Nuclear staining (blue, To-Pro3).

To evaluate the transfer of other organelles between RPTEC we employed a mitochondria specific dye, MitoTracker™ (green). Qtracker 605 labeled cells were co-cultured with MitoTracker labeled RPTEC for 24 h. Due to the limitations of ImageStream or conventional fluorescence microscopy to provide sufficient morphological information of the mitochondria, we employed confocal microscopy and could confirm the presence of mitochondria in the lumen of RPTEC tubes and the spontaneous exchange of these organelles between renal epithelial cells (Figure 4). In addition to mitochondria, we also detected genomic material in a TNT-like structure in RPTEC by DNA-staining (Figure 2C).

## Discussion

We report here the discovery of an unexpectedly high degree of spontaneous intercellular exchange between primary human kidney epithelial cells. Evidence is further provided for the involvement of TNT-like structures in this intercellular communication. Tube based intercellular exchange was only recently discovered to be involved in intercellular communication of immune cells, progenitor cells or tumor cells which together share a high level of cellular motility and genomic plasticity [2]. In contrast, RPTEC and HMEC display a characteristic epithelial cell like tissue architecture i.e., tight cell-cell junctions in culture. Therefore, identification of TNT-like structures and the high level of intercellular exchange was counterintuitive and an intriguing finding.

The diameter of the classical TNTs range between 50–200 nm, they are fragile when exposed to light and they use to connect the cells straight at their nearest distance [1], [2]. In contrast, the here identified tubes are less photo-sensitive e.g., visible under light microscopy as stable structures for a prolonged period of time (up to several minutes). We frequently discovered one or more filopodia- like cell protrusions which may eventually develop into long tubes (up to >200 µm) with relatively large calibers (diameters from 200 nm to >1 µm) as compared to the classical TNTs (Figures 1 and 2). Further, TNTs appear often as straight connections between two cells whereas branched connections between three or more cells are rare [4], [9]. In contrast, we also found bifurcations of the tubes connecting multiple epithelial cells suggesting their potential involvement in communication between these epithelial colonies (Figure 1). Moreover, we observed gondola-like structures along the identified tubes indicating that they might function as transport vehicles.

Induction of cellular stress by treatment of RPTECs with Zeocin dose dependently increased the formation of TNT-like structures. This finding is in alignment with previous reports demonstrating an induction of intercellular communication by cellular stress, for example after treatment of astrocytes with hydrogen peroxide [10]. Likewise, expression and formation of other intercellular channels such as gap junctional communication was reported to be induced after activation of microglia and monocytes/macrophages lineage by various compounds and stress stimuli [11]–[13]. These data indicate that tube-genesis might constitute a coordinated strategy for RPTEC to facilitate intercellular communication in response to cellular stress.

These initial heuristic observations prompted us to investigate the kinetic and extent of potential intercellular exchange in renal epithelial homeostasis. Therefore, we established a co-culturing system of RPTECs labeled with two distinct fluorescent Qdots. Extensive spontaneous exchange of Qdots between RPTECs was detected by fluorescence microscopy (Figure 2). In alignment with the proposed transport vehicle function of the tubes, quantum particles could be detected in the lumen of the intercellular bridges suggesting that at least in part the intercellular exchange was facilitated by these structures (Figure 2). High-throughput fluorescence image analysis revealed that spontaneous intercellular exchange occurred in a substantial fraction of RPTEC (67.5%, Figure 3B). The majority of double positive cells were characterized by presence of only few alternately labeled Qdot organelles suggesting unidirectional transport as the predominant pattern of exchange in RPTECs.

F-actin polymerization was shown to be essential for TNT genesis, stabilization and intra-tubular transport [1], [2], [9]. To confirm our finding that TNT-like structures are involved in RPTEC communication and to exclude other types of intercellular exchange mechanisms such as sequential exo- and endocytosis or phagocytotic events, Qtracker 605 and 525 labeled RPTEC were co-cultured in the presence of the actin polymerization inhibitor LatB. The significant inhibition (>60%) of spontaneous intercellular exchange detected in LatB treated *vs.* control co-cultures demonstrates the importance of TNT-like tubes in transfer of cell organelles between RPTEC. Hence, the formation of the here identified RPTEC tubes, in line with TNTs, seem to be dependent on actin-polymerization.

The discovery of novel TNT cargos is a rapidly evolving field. Most recently, mitochondria, prion proteins and human immunodeficiency virus (HIV) were proposed to be transported through these intercellular channels [2], [4], [7], [14]. We report here potential transfer of endo-/lysosomal organelles (preferentially labeled by Qtrackers), mitochondria (MitoTracker™) and possibly genomic material by the TNT-like structures in RPTEC.

Our data indicate a central role for intercellular communication via exchange of cytosolic material in epithelial cells isolated from proximal renal tubules. This communication was mediated at least in part via tubular structures trafficking cargo vesicles and organelles. The impact of the here described extensive spontaneous exchange of cellular material in renal physiology and in pathological conditions, e.g. in exchange of pathogens such as viruses, genomic material or proteins warrants further investigation. The failure to detect these structures *in-vivo* so far might be related to the lack of specific makers, their fragility when exposed to light, shearing force or chemical fixation [1]–[3]. From our data, one could speculate that these structures might be more frequent in stress situation, e.g., along the denuded basement membrane of injured tubular segments during acute ischemic and/or toxic injury. Therefore, investigation of intercellular exchange and TNT-like tubes might provide novel insights into the remarkable regenerative capacity of the kidney after acute injury [15], [16]. Together, our data suggest an important role for TNT like structures in intercellular communication of renal epithelial cells.

## Methods

### Reagents and Cell Culture

Primary isolated human renal proximal tubular epithelial cells (Clonetics, Lonza, Walkersville, MD) were maintained in REGM™ media (Clonetics) containing Renal Epithelial Cell Basal Medium supplemented with hEGF 0.1%; Hydrocortisone 0.1%; Epinephrine 0.1%; Insulin 0.1%; Triiodothyronine 0.1%; Transferrin 0.1%; Gentamycin/Amphotericin-1000 0.1% and FBS 0.5%. Reduction mammoplasty derived primary human mammary epithelial cells (HMEC, pre-stasis, specification ID: 184 ◊) were obtained from Dr. Martha Stampfer, Human Mammary Epithelial Cell Bank, Berkley Lab, California and detailed information on generation, morphological- and molecular characteristics of HMECs are available online (http://hmec.lbl.gov). HMEC were cultured in a 1∶1 mixture of MM4- and MEGM media as described in http://hmec.lbl.gov. HMEC culture media consists of 50% MM4media (1∶1 mixture of DME/F12, 0.5% FCS, Insulin 10 µg/ml, EGF 5 ng/ml, Hydrocortisone 0.1 µg/ml, Cholera Toxin 1 ng/ml, Triiodothyronine 10^−8^M, Estradiol 10^−9^M, Penicillin/Streptomycin 1%) and 50% MEGM media (Clonetics, Lonza) with the addition of Transferrin 5 µg/ml, Isoproterenol 10^−5^M and BPE 70 µg/ml. Cells were maintained under standard culture conditions. RPTEC were exposed to stress by treatment with Zeocin (Invitrogen) at indicated concentrations. F-actin polymerase inhibitor Latrunculin B (Lat-B) was purchased from Sigma-Aldrich (St. Louis, MO). For co-culture studies, two differentially labeled RPTEC populations (Qtracker 605 and 525) were co-plated in T75 flasks for 24 h at 37°C in the absence or presence of 1.25 µM Lat-B.

### Labeling of Cell Organelles

To trace intercellular exchange of cytoplasmic vesicles, cells were separately labeled with Qtracker® 605 or 525 Labeling Kits (Invitrogen, Carlsbad, CA) to the final concentration of 45 nM according to the manufacturer's protocol. After labeling with specific Qdots, cells were co-cultured for 24 hours. To exclude potential not tube related mechanisms of Qdot transfer e.g., via uptake of cell debris from dead cells containing fluorescent particles, a medium change immediately after attachment of the living cells to the culture dish was performed. This has previously been shown to be sufficient to measure a true endocytic organelle transfer between the cells and exclude exosome-related mechanisms and diffusion through the culture medium [5]. To label mitochondria, cells were incubated with MitoTracker® Green FM (Invitrogen) according to the manufacturer's protocol. MitoTracker passively diffuses across the plasma membrane of life cells and accumulates in active mitochondria.

### Light and Fluorescence Microscopy

For light and fluorescence microscopy cells were plated on coverslips (22×22mm glass, Brain Research Labs., Cambridge, MA). The coverslips were sterilized under UV light for 1 h, placed in 6-well plates (Corning Inc., Corning, NY), and 1 ml ECL cell attachment matrix (20 µg/ml entacin-collagen IV-laminin) (Upstate, Temecula, CA) was added. Plates were placed at 37°C for 1 h, and the ECL removed. Coverslips were washed twice with the according cell media before cells were seeded into the 6-well plates. After co-culturing the media was removed, cells were washed with PBS and then fixed by incubation for 15 min in 4% paraformaldehyde solution at RT. Nuclear (DNA) staining was performed using To-Pro-3 (Molecular Probes, Invitrogen). To-Pro-3 was added to the cells in a final concentration of 2 µM (1∶500 dilution) in 1x PBS and incubated in dark for 15 minutes at RT. Confocal microscopy was performed using Zeiss LSM 510 Meta Confocal Scanning System (Carl Zeiss, Jena, Germany) as previously described [17]. For fluorescence and phase contrast light microscopy Nikon Eclipse TE200 and Nikon Eclipse Ti inverted microscopes were used.

### FACS and ImageStream Analysis

For fluorescence activated cell sorting**,** cells were washed once with PBS, detached with 0.25 mg/ml trypsin EDTA, trypsin neutralizing solution (Lonza) was added and cells were suspended into 15 ml centrifuge tubes, centrifuged at 260 rcf for 5 min. Supernatant was removed and cells were resuspended in 0.2 ml media in 5 ml polypropylene round-bottom tubes (Becton Dickinson, Franklin Lakes, NJ) and placed on ice. Cells were sorted using a MoFlo instrument (DakoCytomation) cell sorter. Unstained cells were used as controls to optimize for size and autofluorescence. High-throughput bright field and fluorescence image analysis was performed using the ImageStream cell analysis system (Amnis, Seattle, WA) [18]. Multiparametric post-acquisition image analysis was done using IDEAS Application© software.
